# Impact of Safe Rock^®^ Minerals, Mineral Fertilizers, and Manure on the Quantity and Quality of the Wheat Yield in the Rice–Wheat Cropping System

**DOI:** 10.3390/plants11020183

**Published:** 2022-01-11

**Authors:** Santosh Ranva, Yudh Vir Singh, Neelam Jain, Ram Swaroop Bana, Ramesh Chand Bana, Gajender K. Aseri, Raghavendra Madar, Shadi Shokralla, Eman A. Mahmoud, Ahmed M. El-Sabrout, Hosam O. Elansary

**Affiliations:** 1 Amity Institute of Biotechnology, Amity University Rajasthan, Jaipur 302003, India; rudransh972013@gmail.com (S.R.); njain1@jpr.amity.edu (N.J.); 2 ICAR–Indian Agricultural Research Institute, New Delhi 110012, India; yvsingh63@gmail.com (Y.V.S.); banajaitpura11@gmail.com (R.C.B.); 3 Amity Institute of Microbial Technology, Amity University Rajasthan, Jaipur 302003, India; gkaseri@jpr.amity.edu; 4 ICAR-Indian Institute of Soybean Research, Khandwa Road, Indore 452001, India; raghavendra4449@gmail.com; 5 Centre for Biodiversity Genomics, University of Guelph, Guelph, ON N1G 2W1, Canada; sshokral@uoguelph.ca; 6 Department of Food Industries, Faculty of Agriculture, Damietta University, Damietta 34511, Egypt; emanmail2005@yahoo.com; 7 Department of Applied Entomology and Zoology, Faculty of Agriculture (EL-Shatby), Alexandria University, Alexandria 21545, Egypt; elsabroutahmed@alexu.edu.eg; 8 Plant Production Department, College of Food & Agriculture Sciences, King Saud University, Riyadh 11451, Saudi Arabia

**Keywords:** wheat, Safe Rock^®^ Minerals, farmyard manure, growth, yield, grain protein

## Abstract

Rice–wheat (RW) rotation is the largest agriculture production system in South Asia with a multifaceted role in maintaining the livelihood of people. The customary practices and indiscriminate use of synthetic fertilizers have culminated in the decline of its productivity and profitability during the past two decades, thus affecting the sustainability of wheat. Safe Rock^®^ Minerals (SRM) is a multi-nutrient rich natural rock mineral with great potential to manage soil degradation, reducing the input of fertilizers, improving soil fertility, and plant health. Thus, a field trial was conducted at the research farm of ICAR—Indian Agricultural Research Institute, New Delhi from 2016 to 2018 to evaluate the impact of Safe Rock^®^ Minerals (SRM) on biometric parameters, productivity, quality, and nutrient uptake by conventional wheat and System of Wheat Intensification (SWI) in the wheat–rice cropping system. The results indicate that SWI performed better in terms of growth, yield, and quality parameters than conventional wheat. Among nutrient management practices; the highest growth, yield, and yield attributes of wheat were achieved with the use of SRM application 250 kg ha^−1^ + 100% Recommended Dose of Fertilizer (RDF). SRM application also increased grain protein content significantly. In conclusion, the integrated use of SRM with organic manures can serve as an eco-friendly approach for sustainable wheat production.

## 1. Introduction

Wheat (*Triticum aestivum* L.) serves as the third most important staple food after rice and maize for the world’s population. In 2017, around 771.7 million tons of wheat was produced globally from its acreage of 218.5 million ha with a mean production of 3.53 t ha^−1^ [1]. In South Asia, wheat is grown in rotation with rice covering around 13.5 million hectares’ area, including 10.5 million hectares in Indo-Gangetic plains [2], and is the kingpin of food security of this region [3,4]. Rice–wheat (RW) rotation is the largest agriculture production system in South Asia and is fundamental to the employment, income, and livelihood of its people [5]. During past two decades, the RW system is showing signs of fatigue because of continuous use of traditional practices and imbalance and indiscriminate use of synthetic fertilizer, which has resulted in yield stagnation and declining factor productivity [6,7,8]. Such emerging challenges lead to doubt over wheat’s sustainability. 

Conventional agronomic-practices in RW systems lack sustainability [7,8], and are also expensive due to requirement of inputs in huge quantities, resulting into depletion of natural resources. Therefore, there is a need to develop more eco-friendly fertilizer management protocols, such as integrated approaches of nutrient management, which may boost the efficacy of fertilizer-use and help to maintain the production of the wheat [9]. To tackle the problems in the rice–wheat system, numerous integrated crop and resource management practices [8,10,11,12] have been seeded under the framework of conservation agriculture (CA) to enhance soil fertility, yield, economic viability, and, thus, leading to environmentally sustainable rice–wheat cropping systems [13] while putting a check on all other bottlenecks of this system. Conservation Agriculture (CA) practices encompass zero tillage (ZT), appropriate crop rotation, and residue management, which can be a better option to conventional agriculture for maintaining soil quality [8,14]. Crop residues act as a barrier between the soil and the open environment, which may have a great role in soil erosion reduction, and soil quality improvement [12,15]. Soil tillage practices greatly influence physico-chemical [13,16,17] and biological [18] properties of soil. ZT in wheat now has wide acceptance on the Indo-Gangetic Plain (IGP) of NW India [19] due to a reduction in depletion of soil carbon and nutrients loss, and the positive impacts on overall efficiency and productivity [20,21] of this practice.

Utilization of organic source of nutrient as a substitute to chemical fertilizers is not only a strategy to achieve economic and ecological sustainability, but it also helps in increasing the productivity [22,23,24]. However, the availability of organic sources for effective nutrient management is not sufficient in India. Therefore, natural rocks and minerals can be sustainable alternatives for efficient organic nutrient management. There are diverse ways to ensure sustainability and preserve soil health. Agro-geology (which pertains to the utilization of rocks for crops) [25] is a multidisciplinary approach of utilization of natural rocks and minerals for strengthening the agro-ecosystems by playing an important role in integrated nutrient management for soil fertility.

**Figure 1:**
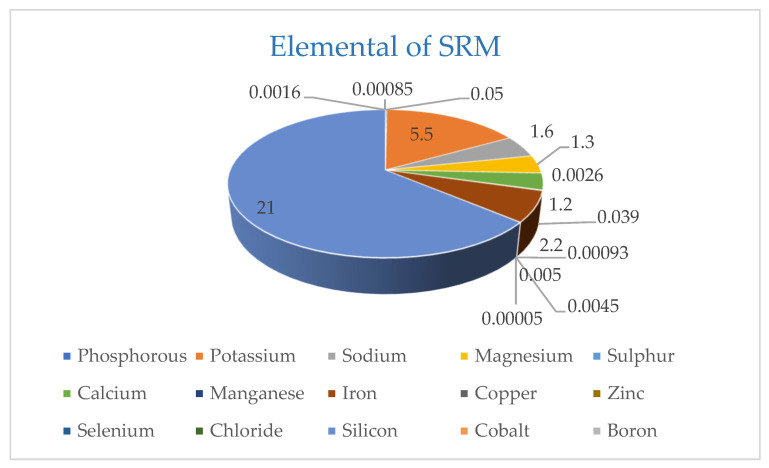
Analysis of Safe Rock^®^ Minerals.

The multi-nutrient rich natural rocks used in fertilization of the soil maintain not only the production levels but also assist in building a sustainable fertile soil through natural processes. This eco-friendly technology facilitates the renewal of the soils and escalates the availability of the necessary macro and micronutrients for the complete development of the plants [26,27]. Safe Rock^®^ Minerals (SRM) is one such option that owns the multi-nutrient natural mineral resource. As per the information received from Safe Rock^®^ Minerals Pvt. Ltd. (New Delhi, India), SRM is an exceptional combination of minerals. Ocean floor south of the Equator was the place from which SRM arose for millions of years. During the pulverization of this hard material into powder form, it produces an exceptional mixture of minerals and nutrients (Figure 1) that are 100% recoverable without any use of additives or the creation of waste. The aim to use SRM is to deliver a genuine product that will lead to eradicating the universal problem of soil degradation and paucity of water. SRM entices and holds Potassium, Ammonium, Calcium, and Magnesium along with other trace elements, which prevent free nutrients from leaching. It is also a mineral soil conditioner that had been authenticated for practice in organic agriculture. The practice of SRM in agricultural/farming will lead to a reduction in the use of chemical fertilizers, as it comprises of numerous minerals and trace elements essential for healthy crops and livestock [28]. This is achieved by its unique balance of nutrients as well as clay minerals, which also increases microbial and earthworm activity and builds long term soil fertility [29]. So far, no scientific information is available on this aspect; therefore, the present study was planned to evaluate the impact of SRM on growth, yield attributes, productivity, and quality of wheat in the RW cropping system.

## 2. Results and Discussion 

### 2.1. Growth Parameters

The application of SRM 250 kg ha^−1^ + 100% RDF produced significantly (*p* < 0.05) higher growth attributes of conventional wheat and SWI, i.e., plant height (91.78 and 92.54; 92.75 and 93.78 cm), tillers m^−2^ (400.33 and 402.78; 404.19 and 407.12), and accumulation of dry matter (32.41 and 34.66; 33.03 and 34.11 g plant^−2^) during 2017 and 2018, respectively, which was significantly superior over only SRM 250 kg ha^−1^ except with dry matter in conventional wheat in 2017, and at par with remaining fertilization treatments. Integration of SRM 250 kg ha^−1^ along with 100% RDF enhanced the growth by 29.72 and 29.54; 24.88 and 24.48% (plant height), 8.17 and 8.24; 8.82 and 8.70% (tillers m^−2^) and 5.71 and 11.02; 15.61 and 15.39% (dry matter) over only SRM application in respective establishment methods and year of experimentation, respectively (Table 1). Moreover, the lowest growth attributes were measured in only SRM 250 kg ha^−1^. Increments in growth values might be due to the increased availability of all essential nutrients due to application SRM and chemical fertilizers. Balanced crop nutrition plays a significant part in rapid cell division and elongation in meristematic plant tissues, growth, photosynthesis, and protein synthesis, which are accountable for the quantitative upsurge in the plant growth [30,31]. Bandyopadhyay et al., 2010 [32] reported that integrated use of 75% NPK and FYM 5 t ha^−1^ or poultry manure 1.5 mg ha^−1^ or phospho-compost 5 mg ha^−1^ to rainy season crops, and 75% NPK to wheat, significantly improved the yield of wheat over-application of 100% NPK in both the season.

### 2.2. Yield Attributes

As per data on yield attributes of the wheat crop in Table 2 and Table 3, all the yield variables were significantly affected during years 2016–17 and 2017–18 due to the different application of plant nutrition. Application of SRM 250 kg ha^−1^ + 100% RDF produced significantly (*p* < 0.05) higher yield attributes of conventional wheat and SWI, viz., number of spikes m^−2^, spike length, spike weight, grains spike^−1^, and test weight, which was 11.15, 10.29, 9.04, and 9.47; 33.48, 32.53, 37.01, and 38.10; 32.75, 32.31, 34.13, and 29.64; 35.54, 37.55, 34.88, and 34.73; and 38.11, 39.02, 58.56, and 52.43 per cent higher than only SRM 250 kg ha^−1^ during 2017 and 2018, respectively. However, the number of spike m^−2^ remained on par with all the treatment except only SRM 250 kg ha^−1^, whereas spike length and spike weight to only No SRM + 100% RDF and significantly (*p* < 0.05) superior to rest of the level. In the case of grains spike^−1^ and test weight recorded at par with all the levels except SRM + 50 % RDF (organic) and only SRM 250 kg ha^−1^ in both the planting techniques. Though, lowest yield attributes were measured in only SRM 250 kg ha^−1^ during years 2016–17 and 2017–18. This may be because of improved inorganic and organic sources, as nutrients were supplied at an adequate rate for a longer duration, which will affect crop growth and photosynthetic activity [33,34]. Integrated use of 75% NPK and FYM 5 t ha^−1^ or poultry manure 1.5 mg ha^−1^ or phospho-compost 5 mg ha^−1^ to rainy season crops, and 75% NPK to wheat, significantly improved the yield of wheat over-application of 100% NPK in both the seasons [35].

### 2.3. Yield and Harvest Index

SRM and inorganic fertilizer’s combined application showed a great impact on stopping the declination in production. The current research reported that grain and straw yields were more in years 2017–2018 as compared to years 2016–2017. The yield as well as harvest indexes showed significant (*p* < 0.05) variation because of an increase in the fertility level and touched the maximum in the application of SRM 250 kg ha^−1^ + 100% RDF (Table 4 and Table 5). Relative to the only SRM 250 kg ha^−1^ treatment, the grain yield increase under SRM 250 kg ha^−1^ + 100% RDF was 36.31 and 35.05% in conventional wheat, and 32.55 and 30.15% in SRI during both the year of study, respectively. Although grain yield remained statistically on par with No SRM + 100% RDF. Next to that, the straw yield, biological yield, and harvest index increased in respective to the treatment relative to only SRM 250 kg ha^−1^, and were on par with all treatment except only SRM 250 kg ha^−1^. The minimum yields and harvest index were recorded in only SRM 250 kg ha^−1^ in both the years of investigation. This might be because, by the application of nutrients through the soil micro-organisms, activity leads to its better utilization, which is involved in nutrient transformation and fixation [36], and also the transportation of nutrients from organic sources affects the nutrient availability to the crop plants as well as the potential for higher manufacture [37]. Grain yield response in wheat is influenced by several agronomic traits [38], such as plant height, number of productive tillers, grain number spike^−1^, spike length (SL), number of kernels spike^−1^, thousand seed weight, grain weight per spike, harvest index (HI), total biomass, and physiological traits [39,40]. Apart from this, SRM provides vital plant nutrients and other growth-promoting substances like enzymes and hormones, which cannot be provided by other artificial fertilizers. Yield enhancement in cereal crops is due to integrated plant nutrient supply and balanced nutrition [23,24,41]. Hence, SRM and inorganic fertilizer’s combined application can make a significant contribution to wheat productivity and sustainability. However, further research is required under long-term experiments as to how the combination affects the soil health in the long-run. Integrated use of 75% NPK and FYM 5 t ha^−1^ or poultry manure 1.5 mg ha^−1^ or phospho-compost 5 mg ha^−1^ to rainy season crops, and 75% NPK to wheat, significantly improved the yield of wheat over-application of 100% NPK in both the season [35]. 

### 2.4. Quality Parameters

The effects of different nutrient management options were significant (*p <* 0.05) on wheat quality parameters (Table 6). Investigation showed significantly (*p* < 0.05) higher milling percent and protein content with application of SRM 250 kg ha^−1^ + 100% RDF, which remained statistically on par with all treatment except the only SRM 250 kg ha^−1^. Compared to only SRM 250 kg ha^−1^, protein content under the respective nutrient option increased by 15.52–21.29% in both the methods of plantation. Moreover, the lowest quality parameter was observed under the only SRM 250 kg ha^−1^. It can be attributed to intense protein synthesis in plants and its efficient storage in the company of a plentiful quantity of accessible nutrients through SRM and inorganic [24]. A balanced C:N ratio was obtained due to nutrients availability, which improved the vegetative growth of plants leading to high photosynthetic activity. The outcome was increased protein content in plant and better yield grain, which subsequently enhanced the protein yield [34,42].

### 2.5. Total N, P, and K Uptake of Wheat

**Figure 2:**
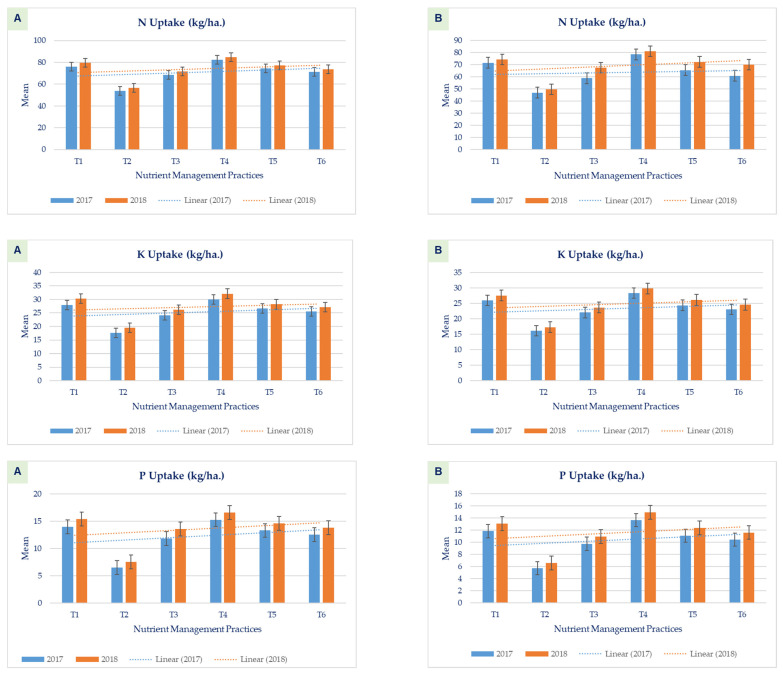
Effect of application of Safe Rock^®^ Minerals on Total N, P, and K uptake in a grain of (**A**) SWI Wheat (**B**) Conventional Wheat.

Total N, P, and K concentrations and uptake in conventional and SWI wheat recorded in 2017 and 2018 were influenced due to different treatments, including SRM applied with other mineral and organic sources of crop nutrition under conventional as well as SWI conditions (Figure 2). The significant (*p* < 0.05) maximum N, P, and K concentrations, and their uptake were recorded with the application of SRM + 100% RDF followed by SRM + 100% RDF, SRM 500 kg ha^−1^ + 75% RDF, and SRM 375 kg ha^−1^ + 75% RDF. The lowest uptake was found with the application of SRM only at 250 kg ha^−1^ at all the observations under the conventional method, as well as SWI wheat. Ranva et al., 2019 [28], have reported a similar pattern of nutrient uptake using SRM in rice crops. Enhancement of growth parameters and nutrient uptake in wheat crops due to the increasing level of K application has also been supported by Ahmad et al., 2008 [43], and Baque et al., 2006 [44], respectively. A higher rate of K promotes efficient use of more nitrogen and also promotes photosynthetic activity, which results in better early vegetative growth and leaf area index [45,46,47]. This is possibly due to the better availability of micronutrient content from Safe Rock^®^ Minerals itself. However, more importantly, the clay minerals within Safe Rock^®^ Minerals efficiently increased the availability of nutrients within the soil, and it increased the availability of nutrients in plants. 

These results provide strong evidence that the dual goals of enhancing productivity and quality of wheat substantially under rice–wheat cropping systems in Indo-Gangetic plains of Northern India can be achieved sustainably with Integrated Nutrient Management [12] using multi-nutrient rich and multifaceted SRM [28] along with organic sources of nutrients.

## 3. Materials and Methods

### 3.1. Description of the Experimental site

**Figure 3:**
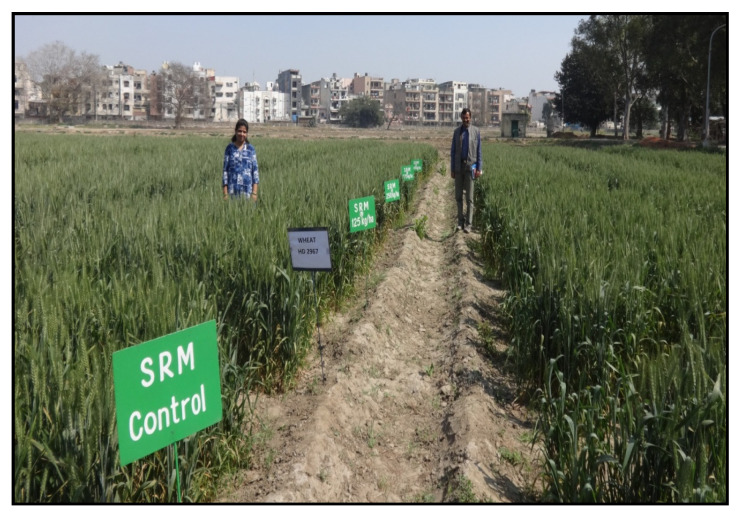
The experimental site at ICAR-IARI.

The current research was performed during *the rabi* seasons of 2016–2017 to 2017–2018 at ICAR—Indian Agricultural Research Institute, New Delhi, India. This site (Figure 3) is situated at an altitude of 228.60 m above mean sea level with a latitudinal longitudinal extent is of 28°40′ N and 77°12′ E, respectively, and has a semi-arid climate. Average annual rainfall of the last 30 years is 652 mm and annual pan evaporation is ~850 mm. The total rainfall received during the experimental seasons was 2.2 mm in 2017 and 4.4 mm in 2018 (Table 7).

### 3.2. Soil Sampling and Analysis

Before the beginning of the experiment, the composite soil sample of the same field was picked up from soil depths of 0–5, 5–15, and 15–30 cm by utilizing using an auger of 5 cm in diameter. The soil samples were air-dried under the shade, ground, and passed across a 2 mm sieve and tested for various Physico-chemical parameters like Electrical conductivity (EC), pH, organic carbon (OC), N, P, K, Sand, Silt, and Clay (Table 8). The experimental soils were found to be sandy loam in texture. 

### 3.3. Experimental Design and Treatments

The design of the trial was a randomized block having amalgamations of six treatments; T_1_: No SRM + 100% RDF, T_2_: Only SRM 250 kg ha^−1^, T_3_: SRM + 50% RDF, T_4_: SRM + 100% RDF, T_5_: SRM + 50% RDF (chemical) + 25% RDF (organic-FYM), and T_6_: SRM + 50% RDF (organic-FYM). Each treatment was taken in both planting method of wheat variety HD-2967 like; Conventional and System of Wheat Intensification (SWI). RDF for NPK nutrient were applied as chemical fertilizer, viz., urea, di-ammonium phosphate and muriate of potash, and organic manure, viz., FYM. Wheat was fertilized with 120 kg N ha^−1^, 60 kg P_2_O_5_ ha^−1^, and 40 kg K_2_O ha^−1^. Further details of nutrient management in each treatment are shown in Table 9 and Figure 1. Statistical analysis was performed with the help of the *F*-test and the level of significance (*p*) was set at <0.05. The treatments included:

General recommendations for the crop were followed for performing all other agronomic practices excluding the treatments. Each treatment was divided into three replications since the plot size was relatively large (12 m^2^). In all the plots of the experiment zero tillage operations were carried out.

### 3.4. Measurement of Growth Parameters

#### 3.4.1. Plant Height (cm) at Maturity

Random selection of ten plants was performed in three places from a square meter area in each experimental unit. These tillers were measured in centimeters and their mean values obtained. 

#### 3.4.2. The Number of Tillers m^−2^

One spot of one square meter were randomly fixed in each plot. The quantity of tiller from each selected spot within the plot was calculated and stated as the number of tillers m^−2^.

#### 3.4.3. Dry Matter Accumulation

Samples of plant for dry matter accumulation were taken from the second and penultimate row at harvesting from each plot. Samples were sundried and then moved into the oven to dry at a temperature of 65 ± 5 °C, so that a constant weight can be attained. The next step was to calculate matter accumulation in g plant^−1^.

### 3.5. Yield Attributes

#### 3.5.1. The Number of Spikes m^−2^

The number of spikes within one-meter square area was recorded at maturity and averaged to arrive at the mean number of spikes m^−1^ row length, and finally they were converted to the number of spikes m^−2^.

#### 3.5.2. Spike Length (cm)

The length of randomly selected ten spikes from the tagged plants of sampling rows was taken, and average data were recorded in cm. 

#### 3.5.3. Spike Weight (g)

The total weight of the randomly selected ten spikes was taken and averaged to arrive at the weight of a spike in grams.

#### 3.5.4. The Number of Grains Per Spike

The total number of grains from the randomly selected ten spikes from the tagged plants of sampling rows were counted and averaged to arrive at the number of grains per spike.

#### 3.5.5. 1000-Grain Weight

One-thousand grains were randomly taken from the bulk production of each net plot, and were counted and weighed. The weight was expressed as 1000-grain weight in grams.

### 3.6. Yield and Harvest Index

#### 3.6.1. Grain Yield (t ha^−1^)

The bundles obtained for recording biological yield were dried under the sun and then with the help of a mechanical thresher, seeds were separated, weighed in kilograms, and then the data were changed into t ha^−1^.

#### 3.6.2. Straw Yield (t ha^−1^)

The total biological yield (grain + straw) from the net plot was recorded and straw yield was worked out by subtracting the grain yield from the biological yield and expressed in t ha^−1^. 

#### 3.6.3. Biological Yield (t ha^−1^)

In each plot, the crop plants were harvested, fastened into bundles separately, and then weighed in kilograms with the help of a spring balance and after that, the data were converted into t ha^−1^. 

#### 3.6.4. Harvest Index (%)

The ratio of economic yield to the biological yield (harvest index) was computed using the following formula:

Harvest index (%) = Economic yield (grains)/Biological yield (grain + straw) × 100

### 3.7. Quality Parameters

#### 3.7.1. Milling (%)

Two-hundred grams of grain from wheat samples were milled with a laboratory mill, and the weight of the milled wheat sample was taken to calculate the percentage of total milled wheat.

Milling (%) = Wt. of milled wheat (g)/Wt. of rough wheat (g) × 100

#### 3.7.2. Protein Content (%)

Protein contents of wheat seed were estimated by micro Kjeldahl digestion to determine nitrogen content, which were then converted to protein by multiplying respective nitrogen content in wheat grain with a factor 6.25 [54].

### 3.8. N, P, and K Concentration in Grain at Harvesting

N content (%), Phosphorus (%), and Potassium (%) content in grain were recorded by the modified Kjeldahl method, Vanado–molybdophosphoric acid yellow color method, and Flame photometer method [48], respectively. 

### 3.9. Statistical Analysis

All the data obtained from the experiment conducted under factorial randomized block design were statistically analyzed using the *F*-test as per the procedure given by Gomez and Gomez, 1984 [55]. LSD values at *p* = 0.05 were used to determine the significance of the difference between treatment means. The treatment effects were tested at 5% probability level for their significance.

## 4. Conclusions

Rice–wheat cropping system is a base for the food and nutritional security of South-Asian countries. From the current study, it was found that for sustaining the productivity of this system, the recommended dose of fertilizer may be supplemented with Safe Rock^®^ Minerals (SRM) 250 kg ha^−1^. The crop supplemented through SRM nutrition also produces a better quality of wheat grain, which may help fight against hunger and malnourishment in the irrigated agroecosystems of India and neighboring regions. Considering all the parameters, it was shown that the integrated nutrient management using 250 kg ha^−1^ SRM + 50% RDF through chemical fertilizer, and the remaining 25% RDF with FYM, is a sustainable option for enhancing the productivity and quality of wheat and augmenting the soil health status of these regions too. Future research may focus on the long-term effect of SRM on soil health in diverse ecologies and on the nutrient release patterns of the combinations in different farm typologies.

## Figures and Tables

**Table 1:** Effect of Safe Rock^®^ Minerals application on growth parameters of wheat in the rice–wheat cropping system.

Treatments	Plant Height (cm)				Tillers m^−2^				Dry Matter (g Plant^−1^)			
	Conventional Wheat		SWI		Conventional Wheat		SWI		Conventional Wheat		SWI	
	2017	2018	2017	2018	2017	2018	2017	2018	2017	2018	2017	2018
No SRM + 100% RDF	88.44 ^a^	88.56 ^a^	90.18 ^a^	92.12 ^a^	397.40 ^a^	399.56 ^a^	398.66 ^a^	401.76 ^a^	32.27 ^a^	33.45 ^ab^	32.46 ^a^	33.55 ^a^
Only SRM 250 kg ha^−1^	70.75 ^b^	71.43 ^b^	74.27 ^b^	75.34 ^b^	370.11 ^b^	372.12 ^b^	371.44 ^b^	374.53 ^b^	30.66 ^a^	31.22 ^b^	28.57 ^b^	29.56 ^b^
SRM + 50% RDF	84.62 ^a^	85.34 ^a^	86.32 ^a^	87.56 ^a^	392.32 ^ab^	395.67 ^ab^	395.12 ^ab^	396.23 ^ab^	31.95 ^a^	32.89 ^ab^	31.78 ^a^	32.97 ^a^
SRM + 100% RDF	91.78 ^a^	92.54 ^a^	92.75 ^a^	93.78 ^a^	400.33 ^a^	402.78 ^a^	404.19 ^a^	407.12 ^a^	32.41 ^a^	34.66 ^a^	33.03 ^a^	34.11 ^a^
SRM + 50% RDF (chemical) + 25%RDF (organic-FYM)	86.85 ^a^	87.45 ^a^	89.22 ^a^	91.78 ^a^	396.41 ^a^	398.56 ^a^	397.33 ^a^	400.54 ^ab^	32.17 ^a^	33.32 ^ab^	31.98 ^a^	33.34 ^a^
SRM + 50% RDF (organic-FYM)	86.16 ^a^	87.21 ^a^	88.24 ^a^	90.23 ^a^	395.27 ^ab^	397.34 ^ab^	396.66 ^ab^	397.23 ^ab^	32.01 ^a^	33.12 ^ab^	31.84 ^a^	33.12 ^a^
* SEm±	2.12	2.34	2.45	2.52	8.11	8.32	8.24	8.31	0.91	0.95	1.14	1.21
** LSD (*p* = 0.05)	7.17	7.24	7.78	7.82	25.66	26.31	25.87	26.08	2.87	2.94	3.11	3.22

* SEm±: Standard Error of Mean, ** LSD: Least Significant Difference; Means followed by a similar lowercase letter within a column are not significantly different (at *p* < 0.05) according to Tukey’s HSD test.

**Table 2:** Effect of Safe Rock^®^ Minerals application on yield attributes of conventional wheat in the rice–wheat cropping system.

Treatment	2017					2018				
	No. of Spike m^−2^	Spike Length (cm)	Spike Weight (g)	Grains Spike^−1^	Test Weight (g)	No. of Spike m^−2^	Spike Length (cm)	Spike Weight (g)	Grains Spike^−1^	Test Weight (g)
No SRM + 100% RDF	334.6 ^a^	11.55 ^ab^	3.61 ^ab^	65.11 ^ab^	39.03 ^ab^	336.6 ^a^	11.75 ^ab^	3.75 ^a^	67.56 ^ab^	40.55 ^ab^
Only SRM 250 kg ha^−1^	304.7 ^b^	9.32 ^c^	2.84 ^d^	50.34 ^c^	29.91 ^c^	309.9 ^b^	9.56 ^c^	2.94 ^c^	51.45 ^c^	30.68 ^c^
SRM + 50% RDF	330.7 ^a^	11.09 ^b^	3.25 ^c^	60.12 ^b^	36.71 ^b^	332.8 ^ab^	11.26 ^b^	3.35 ^b^	63.88 ^b^	37.56 ^b^
SRM + 100% RDF	338.7 ^a^	12.44 ^a^	3.77 ^a^	68.23 ^a^	41.31 ^a^	341.8 ^a^	12.67 ^a^	3.89 ^a^	70.77 ^a^	42.65 ^a^
SRM + 50% RDF (chemical) + 25%RDF (organic-FYM)	333.7 ^a^	11.45 ^b^	3.51 ^b^	63.16 ^ab^	38.22 ^ab^	335.8 ^a^	11.67 ^b^	3.67 ^ab^	66.98 ^ab^	39.55 ^ab^
SRM + 50% RDF (organic-FYM)	331.7 ^a^	11.20 ^b^	3.48 ^b^	61.78 ^b^	37.35 ^b^	334.3 ^ab^	11.52 ^b^	3.52 ^b^	64.98 ^b^	38.52 ^b^
* SEm±	7.11	0.28	0.07	1.73	1.03	7.13	0.29	0.08	1.75	1.06
** LSD (*p* = 0.05)	23.56	0.92	0.21	5.13	3.22	24.56	0.94	0.22	5.17	3.25

* SEm±: Standard Error of Mean, ** LSD: Least Significant Difference; Means followed by a similar lowercase letter within a column are not significantly different (at *p* < 0.05) according to Tukey’s HSD test.

**Table 3:** Effect of Safe Rock^®^ Minerals application on yield attributes of SWI in the rice–wheat cropping system.

Treatment	2017					2018				
	No. of Spike m^−2^	Spike Length (cm)	Spike Weight (g)	Grains Spike^−1^	Test Weight (g)	No. of Spike m^−2^	Spike Length (cm)	Spike Weight (g)	Grains Spike^−1^	Test Weight (g)
No SRM + 100% RDF	339.3 ^a^	12.17 ^a^	3.74 ^ab^	67.35 ^ab^	40.12 ^a^	343.0 ^a^	12.51 ^ab^	3.83 ^ab^	69.76 ^ab^	42.13 ^a^
Only SRM 250 kg ha^−1^	312.3 ^b^	9.24 ^c^	2.93 ^c^	50.66 ^c^	26.59 ^c^	315.6 ^b^	9.45 ^c^	3.07 ^c^	52.87 ^c^	28.65 ^c^
SRM + 50% RDF	335.4 ^ab^	11.76 ^b^	3.36 ^b^	63.06 ^b^	35.82 ^b^	339.8 ^ab^	11.88 ^b^	3.54 ^b^	65.87 ^b^	37.86 ^b^
SRM + 100% RDF	340.6 ^a^	12.66 ^a^	3.93 ^a^	68.33 ^a^	42.16 ^a^	345.4 ^a^	13.05 ^a^	3.98 ^a^	71.23 ^a^	43.67 ^a^
SRM + 50% RDF (chemical) + 25%RDF (organic-FYM)	337.3 ^a^	11.94 ^b^	3.59 ^b^	65.45 ^ab^	39.37 ^a^	341.9 ^a^	12.16 ^ab^	3.67 ^b^	68.67 ^ab^	41.23 ^a^
SRM + 50% RDF (organic-FYM)	335.7 ^ab^	11.87 ^b^	3.49 ^b^	63.66 ^ab^	38.88 ^ab^	340.4 ^ab^	12.03 ^b^	3.62 ^b^	67.43 ^ab^	40.76 ^ab^
* SEm±	7.23	0.21	0.08	1.82	1.12	7.31	0.32	0.09	1.84	1.26
** LSD (*p* = 0.05)	24.55	0.66	0.27	5.23	3.31	25.23	0.99	0.28	5.25	3.34

* SEm±: Standard Error of Mean, ** LSD: Least Significant Difference; Means followed by a similar lowercase letter within a column are not significantly different (at *p* < 0.05) according to Tukey’s HSD test.

**Table 4:** Effect of Safe Rock^®^ Minerals application on yields and harvest index of Conventional wheat in the rice–wheat cropping system.

Treatment	2017				2018			
	Grain Yield (t ha^−1^)	Straw Yield (t ha^−1^)	Biological Yield (t ha^−1^)	Harvest Index (%)	Grain Yield (t ha^−1^)	Straw Yield (t ha^−1^)	Biological Yield (t ha^−1^)	Harvest Index (%)
No SRM + 100% RDF	4.56 ^ab^	8.26 ^a^	12.82 ^a^	35.56 ^ab^	4.67 ^ab^	8.37 ^a^	13.04 ^a^	35.81 ^a^
Only SRM 250 kg ha^−1^	3.58 ^c^	7.12 ^b^	10.72 ^b^	33.45 ^b^	3.68 ^c^	7.26 ^b^	10.94 ^b^	33.63 ^b^
SRM + 50% RDF	4.24 ^b^	7.85 ^a^	12.09 ^a^	35.07 ^ab^	4.38 ^b^	7.98 ^a^	12.36 ^a^	35.43 ^a^
SRM + 100% RDF	4.88 ^a^	8.34 ^a^	13.22 ^a^	36.91 ^a^	4.97 ^a^	8.23 ^a^	13.21 ^a^	37.65 ^a^
SRM + 50% RDF (chemical) + 25%RDF (organic-FYM)	4.43 ^b^	8.15 ^a^	12.58 ^a^	35.21 ^ab^	4.58 ^b^	8.15 ^a^	12.73 ^a^	35.97 ^a^
SRM + 50% RDF (organic-FYM)	4.35 ^b^	8.01 ^a^	12.36 ^a^	35.19 ^ab^	4.47 ^b^	7.78 ^ab^	12.25 ^a^	36.48 ^a^
* SEm±	0.11	0.18	0.37	0.68	0.13	0.19	0.38	0.69
** LSD (*p* = 0.05)	0.35	0.66	1.13	2.23	0.36	0.67	1.15	2.25

* SEm±: Standard Error of Mean, ** LSD: Least Significant Difference; Means followed by a similar lowercase letter within a column are not significantly different (at *p* < 0.05) according to Tukey’s HSD test.

**Table 5:** Effect of Safe Rock^®^ Minerals application on yields and harvest index of SWI in the rice–wheat cropping system.

Treatment	2017				2018			
	Grain Yield (t ha^−1^)	Straw Yield (t ha^−1^)	Biological Yield (t ha^−1^)	Harvest Index (%)	Grain Yield (t ha^−1^)	Straw Yield (t ha^−1^)	Biological Yield (t ha^−1^)	Harvest Index (%)
No SRM + 100% RDF	4.82 ^ab^	8.43 ^a^	13.25 ^a^	36.37 ^ab^	4.97 ^ab^	8.54 ^a^	13.51 ^a^	36.78 ^a^
Only SRM 250 kg ha^−1^	3.84 ^c^	7.29 ^b^	11.13 ^b^	34.50 ^b^	3.98 ^c^	7.35 ^b^	11.33 ^b^	35.12 ^a^
SRM + 50% RDF	4.57 ^b^	7.97 ^ab^	12.54 ^a^	36.42 ^ab^	4.68 ^b^	8.26 ^a^	12.94 ^a^	36.16 ^a^
SRM + 100% RDF	5.09 ^a^	8.59 ^a^	13.68 ^a^	37.20 ^a^	5.18 ^a^	8.68 ^a^	13.86 ^a^	37.37 ^a^
SRM + 50% RDF (chemical) + 25%RDF (organic-FYM)	4.75 ^ab^	8.22 ^a^	12.97 ^a^	36.62 ^ab^	4.78 ^b^	8.47 ^a^	13.33 ^a^	36.45 ^a^
SRM + 50% RDF (organic-FYM)	4.65 ^b^	8.11 ^a^	12.76 ^a^	36.44 ^ab^	4.73 ^b^	8.34 ^a^	13.11 ^a^	36.33 ^a^
* SEm±	0.12	0.21	0.38	0.71	0.13	0.24	0.39	0.74
** LSD (*p* = 0.05)	0.38	0.71	1.17	2.22	0.39	0.74	1.19	2.29

* SEm±: Standard Error of Mean, ** LSD: Least Significant Difference; Means followed by a similar lowercase letter within a column are not significantly different (at *p* < 0.05) according to Tukey’s HSD test.

**Table 6:** Effect of Safe Rock^®^ Minerals application on different quality parameters of wheat in the rice–wheat cropping system.

Treatment	Conventional Wheat				SWI			
	2017		2018		2017		2018	
	Milling %	Protein Content (%)	Milling %	Protein Content (%)	Milling %	Protein Content (%)	Milling %	Protein Content (%)
No SRM + 100% RDF	67.66 ^a^	9.34 ^a^	68.66 ^a^	9.46 ^a^	68.33 ^a^	9.40 ^a^	69.18 ^a^	9.52 ^a^
Only SRM 250 kg ha^−1^	57.87 ^b^	7.89 ^b^	58.54 ^b^	8.03 ^b^	58.45 ^b^	8.33 ^b^	59.76 ^b^	8.44 ^a^
SRM + 50% RDF	64.18 ^a^	8.95 ^a^	65.99 ^a^	9.16 ^a^	65.23 ^a^	8.98 ^ab^	66.98 ^a^	9.10 ^ab^
SRM + 100% RDF	69.04 ^a^	9.57 ^a^	70.09 ^a^	9.69 ^a^	69.33 ^a^	9.63 ^a^	69.88 ^a^	9.75 ^a^
SRM + 50% RDF (chemical) + 25%RDF (organic-FYM)	66.66 ^a^	9.20 ^a^	67.99 ^a^	9.40 ^a^	67.33 ^a^	9.34 ^a^	68.55 ^a^	9.46 ^a^
SRM + 50% RDF (organic-FYM)	65.66 ^a^	9.05 ^a^	66.87 ^a^	9.34 ^a^	66.66 ^a^	9.10 ^a^	67.98 ^a^	9.22 ^a^
* SEm±	1.83	0.20	1.85	0.23	2.34	0.21	2.38	0.23
** LSD (*p* = 0.05)	5.56	0.64	5.58	0.69	6.45	0.67	6.51	0.69

* SEm±: Standard Error of Mean, ** LSD: Least Significant Difference; Means followed by a similar lowercase letter within a column are not significantly different (at *p* < 0.05) according to Tukey’s HSD test.

**Table 7:** Monthly variations in weather conditions during October 2017 to May 2018.

Month	T. Max. (°C)		T. Min. (°C)		Wind Speed (kmph)		Total Rainfall (mm)		Total Evaporation (mm)		BSS (h)	
	2017	2018	2017	2018	2017	2018	2017	2018	2017	2018	2017	2018
October	33.9	34.0	16.2	17.2	3.2	2.4	1.2	0.0	4.8	3.9	5.2	6.2
November	29.0	26.8	9.0	10.5	3.1	2.3	0.0	0.0	3.8	2.3	2.8	2.6
December	23.3	23.0	5.2	6.8	3.0	3.1	0.0	0.2	2.9	2.3	4.6	4.3
January	20.7	20.1	6.5	7.7	3.2	4.0	0.0	2.1	2.5	2.2	2.4	4.1
February	24.6	24.1	8.1	9.8	4.3	4.9	0.0	0.0	3.1	4.1	6.0	7.1
March	30.8	29.7	13.7	13.9	5.3	5.5	0.6	0.6	5.3	5.3	6.8	8.1
April	38.7	38.4	19.1	20.7	6.9	6.5	0.0	0.3	8.2	6.9	7.8	8.6
May	40.1	39.8	23	24.3	7.1	5.6	1.0	1.2	8.9	6.5	7.5	7.7

(T. Max.: Maximum Temperature, T. Min.: Minimum Temperature, BSS: Bright Sunshine).

**Table 8:** Physicochemical properties of soil at the experiment site.

Parameters	Value	Methods Employed
**Chemical Properties**		
pH	8.33	1:2.5 Soil water suspension measured with Glass electrode pH [48]
EC (dS m^−1^)	0.37	1:2.5 Soil water suspension measured with Systronics conductivity meter [48]
OC (g kg^−1^ soil)	0.43	Walkley and Black method [49]
Available N (kg ha^−1^)	143	Alkaline Potassium Permagnate method [50]
Available P (kg ha^−1^)	13.45	Sodium Bicarbonate method [51]
Available K (kg ha^−1^)	245	Ammonium acetate method [52]
**Mechanical Properties**		
Sand (%)	57.63	Bouyoucous Hydrometer method [53]
Silt (%)	22.92	
Clay (%)	19.52	
Textural Class	Sandy loam	

**Table 9:** Nutritional composition of Chemical and organic source of fertilization.

Nutrients	Chemical Fertilizer			Organic Manure
	Urea (%)	Di-Ammonium Phosphate (%)	Muriate of Potash (%)	FYM (%)
Nitrogen	46	-	-	0.59
Phosphorous	18	46	-	0.22
Potassium	-	-	60	0.53
Calcium	-	-	-	0.93
Magnesium	-	-	-	0.21
Iron	-	-	-	146.5 ppm
Manganese	-	-	-	69.4 ppm
Zinc	-	-	-	15.8 ppm
Copper	-	-	-	2.7 ppm

## Data Availability

All data are available in this publication.
